# Seven core competencies and conditions for equitable partnerships and power sharing in community-based participatory research

**DOI:** 10.1136/bmjgh-2024-015497

**Published:** 2024-11-17

**Authors:** Kim Ozano, Wafa Alam, Bachera Aktar, Linet Okoth, Ivy Chumo, Jessica Amegee Quach, Nelly Muturi, Samuel Saidu, Ibrahim Gandi, Neele Wiltgen Georgi, Lilian Otiso, Abu Conteh, Sally Theobald, Laura Dean, Rachel Tolhurst, Robinson Karuga, Jiban Karki, Surekha Garimella, Vinodkumar Rao, Anthony Mwanki, Nazia Islam, Sia Morenike Tengbe, Sweta Dash, Prasanna Subramanya Saligram, Sabina Rashid, Rosie Steege

**Affiliations:** 1 Public Health, Liverpool School of Tropical Medicine, Liverpool, UK; 2 BRAC University James P Grant School of Public Health, Dhaka, Bangladesh; 3 Liverpool School of Tropical Medicine, Liverpool, UK; 4 LVCT, Nairobi, Kenya; 5 Research Division, APHRC, Nairobi, Kenya; 6 NCD Alliance, Geneva, Switzerland; 7 International Rescue Committee, Nairobi, Kenya; 8 COMAHS, Freetown, Western Area, Sierra Leone; 9 CODOHSAPA/FEDURP, Freetown, Sierra Leone; 10 Department of International Public Health, Liverpool School of Tropical Medicine, Liverpool, UK; 11 Liverpool VCT Care and Treatment, Nairobi, Kenya; 12 SLURC, Freetown, Sierra Leone; 13 The George Institute for Global Health India, New Delhi, India; 14 Slum Dwellers International, Cape Town, South Africa; 15 BRAC, Dhaka, Bangladesh; 16 Sierra Leone Ministry of Health and Sanitation, Freetown, Sierra Leone

**Keywords:** Global Health, Health policy, Health services research, Accountability, Qualitative study

## Abstract

Equitable health research requires actively engaging communities in producing new knowledge to advocate for their health needs. Community-based participatory research (CBPR) relies on the coproduction of contextual and grounded knowledge between researchers, programme implementers and community partners with the aim of catalysing action for change. Improving coproduction competencies can support research quality and validity. Yet, frameworks and guidance highlighting the ideal competencies and conditions needed for all research partners to contribute meaningfully and equitably are lacking. This paper aims to advance CBPR by laying out seven core competencies and conditions that can promote power sharing in knowledge production, application and dissemination at the individual, community, organisational and systems levels.

Competencies were developed through an iterative process, that synthesised pre-existing literature and frameworks with a wide range of tacit knowledge from researchers, activists, implementation partners and community researchers from Bangladesh, India, Kenya, Sierra Leone and the UK.

The seven core competencies and conditions are: (1) capacity to interpret and respond to individual and relational identity, connection, uniqueness and inequities; (2) ability of communities and partners to work in the most suitable, inclusive and synergistic way; (3) aptitude for generating safe and inclusive spaces for multidirectional knowledge and skills exchange that goes beyond the research focus; (4) expertise in democratic leadership and/or facilitation to balance competing priorities and ensure shared decision-making; (5) capacity to analyse readiness for action, successes and areas for improvements throughout the research process; (6) ability to instigate sustainable change processes within the political dimensions of systems, policies and practices using advocacy, lobbying or activism approaches and (7) skills to interpret and disseminate findings and outputs that are understandable, respectful and promote community ownership. We present core competency and condition areas, individual and collective expertise associated with competencies, likely outcomes, examples of activities and sources of evidence.

**WHAT IS ALREADY KNOWN ON THIS TOPIC**

- Coproduction research approaches that place communities at the centre of research partnerships are critical to equitable global health research and decolonising research.
- Coproduction approaches become more inclusive when there is a conscious examination and strengthening of community, academic and associated stakeholder needs and capabilities.
- Frameworks and guidance that highlight the ideal competencies and conditions needed for both community and external implementation/research partners to contribute meaningfully and equitably are lacking.
- Guidance is urgently required to support effective global health partnerships to be reflective of both the sociopolitical, cultural and economic conditions and the ‘hard’ and ‘soft’ skills necessary for meaningful and impactful coproduction in health research.

**WHAT THIS STUDY ADDS**

- This study draws on existing literature, frameworks, and embedded and tacit knowledge from members of the ‘The Accountability and Responsiveness in Informal Settlements for Equity’ research consortium to set out seven core competencies and conditions that community-based participatory research programmes could use to strengthen ‘soft’ and ‘hard’ skills of all partners for more trusting, equitable partnerships.

**HOW THIS STUDY MIGHT AFFECT RESEARCH, PRACTICE OR POLICY**

- This research aims to equip community-based research partnerships with the ability to discuss, critique, plan and embed regular critical exploration of competencies and conditions that exist within partnerships for multidirectional sharing of knowledge and skills, as well as to identify gaps that could hinder research action for change.
- Having the foresight and practical tools to assess competencies and conditions will enable partnerships to build the necessary resources and time to respond to individual, community, organisational, sociopolitical, cultural and economic conditions for sustainable community-led change.

## Background

 Global health research partnerships are often characterised by unequal power dynamics and colonial legacies which limit some partners’ meaningful participation.^1^ To counter this, decolonisation efforts seek to recognise non-Western, localised forms of knowledge and authority, acknowledge discrimination, and disrupt colonial structures and legacies that affect equity.^2^ Coproduction research can play a significant role in decolonising health research by redressing power imbalances, strengthening local capacities and promoting equity.^1^ Community-based participatory research (CBPR) is a coproduction approach that works ‘with’ communities and key actors within a partnership to translate innovation to policy and practice in a way that considers context and centres the voices of people impacted.^3^ However, central to the CBPR approach and equitable partnerships is the opportunity to learn, reflect and adapt. While there has been a recent explosion in the development of numerous ‘principles’ and ‘frameworks’ aimed at guiding global research partnerships towards more equitable, just and fair systems,^4^,^6^ practical steps towards understanding what competencies and conditions are needed for successful CBPR, and how to address and measure improvements in skills over time are lacking.^1 7^ Further, many of these frameworks are aimed at supporting equity between institutions and miss the community lens.^1^

CBPR has its own set of distinct principles and values, centred on collaboration, equitable partnerships, knowledge democracy, shared power, learning and embedded action.^8 9^ Research has demonstrated that CBPR approaches become more inclusive when there is a conscious examination of community, academic and associated stakeholder needs and capabilities.^8 10 11^ Without explicit consideration of both the sociopolitical, cultural and economic conditions and competencies needed for equal leadership and shared decision-making across all partners, power imbalances can remain or become further entrenched.^4 12^ The localised nature of CBPR and the heterogeneity of competency development initiatives means it is difficult to identify generalisable competency needs and evaluate effectiveness.^13^,^17^ However, there are common ‘soft’ and ‘hard’ competencies that have been reported at the project level from a variety of perspectives and theories.^13 14 18^ Tembo *et al*^19^ argue that identifying, communicating and addressing the competencies and conditions required across CBPR partnerships will minimise resistance, distrust and unrealistic expectations from the community, improving the quality of research and outcomes. Further, improving competencies is an effective way of attaining research quality and validity.^13^ Yet, strengthening health research coproduction capacity is a relatively neglected area of work.^9 12 20^

Coproduction competencies extend beyond simply exchanging knowledge about research techniques.^12^ For example, growing coproduction capacity requires creating space for and allowing a diversity of knowledge and expertise and paying attention to power dynamics between team members.^12 20^ Developing competencies also requires medium-term to long-term rather than short-term efforts to establish and institutionalise a culture of health research coproduction across three levels—individual, institutional and contextual.^12^ Further, skills are developed and implemented within the structural limitations created by systemic injustices, and therefore, it is important to consider these when strengthening capacity.^1214 21^,^23^

### Aim

The global interest in coproduction research paradigms is evident in funding guidelines, podcasts and publications.^9 24 25^ Yet, frameworks and guidance that highlight the competencies and conditions needed for all partners to contribute in a meaningful and inclusive way are lacking.^22^ More evidence on the competencies and skills, who needs them and how to promote and track their development is essential if the quality standards of coproduced research are to be realised.^11^ We sought to develop specific competencies and conditions to support fair and just coproduction within CBPR partnerships at different levels (individual, community, organisational and health systems) to fill a critical knowledge gap and contribute to the science of community knowledge to action. Table 1 provides definitions and our interpretations of key terminology used in this manuscript.

**Table 1:** Key definitions

Terminology	Definition
Coproduction	Coproduction is a collaborative model of research that includes stakeholders such as patients, the public, donors, clinicians, service providers and policy-makers. It is a sharing of power, with stakeholders and researchers working together to develop the agenda, design and implement the research, and interpret, disseminate and implement the findings.^9^
Community-based participatory research (CBPR)	A collaborative research approach that equitably involves all partners in the research process and aims to combine knowledge with action to achieve sustainable, social change.^8 29^ A cyclical, iterative process that includes learning research skills and how they can be applied in a local setting while centralising community knowledge and cultures through multidirectional teaching and learning practices.
Community researchers (or community co-researchers)	People directly impacted by the research focus and have an active role in the ‘research partnership’. They are likely to be involved in setting or refining the research agenda, codesigning the research process and collecting and analysing data for social change.^36^
Competence	Competence is the ability to integrate and apply contextually appropriate knowledge, skills and psychosocial factors (e.g. beliefs, attitudes, values and motivations) to best participate within a specified domain or role.^87^
Conditions	The sociopolitical, environmental, cultural and economic contexts in which CBPR takes place.
Soft and hard skills	Soft skills could include confidence, self-esteem, effective leadership or communication capacity for people who are less experienced in research or social advocacy.^18^ These skills have intrinsic value as social empowerment, which can lead to a personal and collective purpose to use coproduced knowledge, to take action and therefore have a sustainable impact on the research aspirations and beyond.^43^ Hard skills could include technical-based skills like understanding legal frameworks that stand to enable action.
Power	Power may be understood as people’s abilities to affect outcomes relevant to their lives.^88^ This may include ‘power over’ others but also ‘power to’ act in one’s interests and ‘power with’ others.^89^ Power is dynamic, relational and exercised in daily life through social practice, drawing on a range of unequally distributed historically and contextually specific social, economic, institutional and political resources.^90^

## Methods

### Project setting and research aim

**Figure 1:**
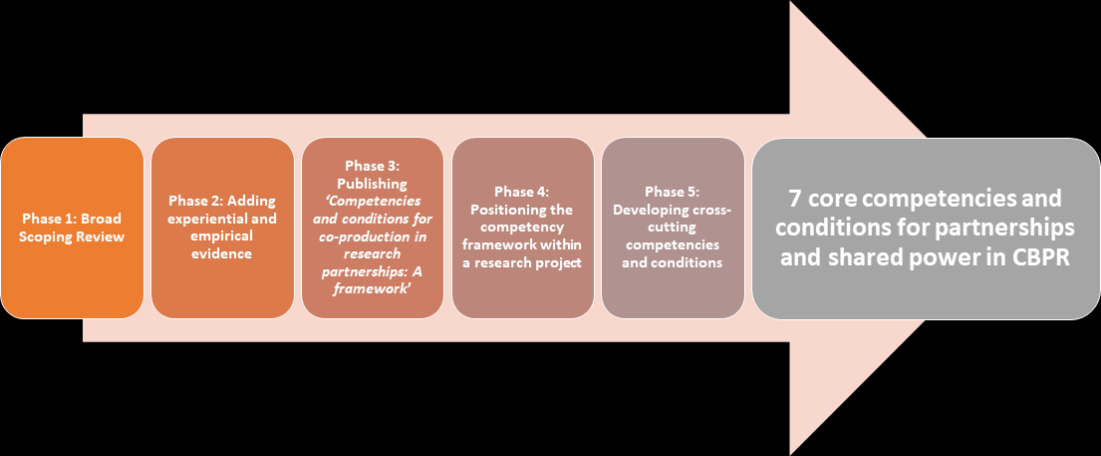
Five steps to developing core competencies and conditions for CBPR partnerships. CBPR, community-based participatory research.

The Accountability and Responsiveness in Informal Settlements for Equity (ARISE) is a multicountry research consortium that uses CBPR to support marginalised residents to analyse their health and well-being and demand their rights to health. ARISE applies CBPR to coproduce knowledge for action through capacity strengthening of people living and working in informal spaces, researchers and implementing partners (non-government and community organisations who are implementing actions at the local level) in Kenya, Sierra Leone, India and Bangladesh. We brought together literature and experiential knowledge of local and global researchers, activists, implementation partners and community researchers involved in ARISE, across countries, through a five-step process to develop our coproduction competencies for action (figure 1).

### Phase 1: a scoping review

We conducted a scoping review, as an iterative knowledge synthesis approach to identify and synthesise existing and emerging literature on frameworks and programmes for competency development.^26^ We mapped this literature against eleven CBPR principles (box 1), developed in 1998 by Israel *et al* and evolved by others.^27 28^ The eleven CBPR principles are positioned as being on a continuum towards an ideal goal.^28^ The extent to which any research effort achieves any combination of these principles will vary depending on context, purpose and participants involved.^29^

Box 1 **Principles of community-based participatory research by Minkler *et al*^8^ and Minkler and Wallerstein^27^**

1. Recognises communities as a unit of identity.
2. Builds on strengths and resources in the community.
3. Facilitates collaborative, equitable partnerships in all research phases and involves an empowering and power-sharing process that attends to social inequalities.
4. Promotes colearning and capacity building among all partners.
5. Integrates and achieves a balance between research and action for the mutual benefit of all partners.
6. Emphasises public health problems of local relevance and ecological perspectives that attend to the multiple determinants of health and disease.
7. Involves systems development through a cyclical and iterative process.
8. Disseminates findings and knowledge gained to all partners and involves all partners in the dissemination process.
9. Requires a long-term process and commitment to sustainability.
10. Openly addresses issues of race, ethnicity, racism and social class and embraces ‘cultural humility’.
11. Works to ensure research rigour and validity but also seeks to ‘broaden the bandwidth of validity’ with respect to research relevance.

The initial literature search took place between 2019 and 2020 as this was the time frame in which the working group of ARISE was focused on understanding competency needs. As the working framework (described below) evolved, additional literature was added by the ARISE consortium until 2023. The initial search strategy included a set of keywords on participatory and coproduction research, capacity strengthening and community engagement. Following the initial selection of 34 articles, article content was analysed by a group of five reviewers (KO, WA, JAQ, BA, LO) from across the five country contexts of ARISE. We used a framework analysis approach to interpret and structure data using the eleven CBPR principles as priori themes to code data using NVivo V.12.^30^ The framework approach described by Gale *et al*^31^ included the reviewers reading through the literature to become familiar with the content, then coding the content to the most appropriate CBPR principle using line-by-line coding, sharing and discussing coding techniques to ensure multiple perspectives were considered. Finally, all coded data were extracted and synthesised for each principle to develop a working framework.^31^

### Phase 2: adding experiential and empirical evidence

The working framework was then presented during a two-hour online ARISE consortium meeting where experiential and empirical evidence was sought verbally, in the chat box function and using an online participation tool for anonymous input. ARISE members were also invited to provide written feedback on the Word version and were encouraged to share with community researchers for discussion. Review and additions to each of the 11 CBPR principles were added based on experiential knowledge of ARISE partners and additional literature. This process was conducted on two separate occasions to gain input at different stages of iteration.

### Phase 3: adaptation and development of the ‘Competencies and conditions for co-production in research partnerships: A framework’

Based on feedback from consortium members and community researchers, the language of the framework was adapted to be more accessible and to align with cross-country partners’ experience. The final format and content were agreed by a CBPR-focused subgroup and an online version of a framework entitled ‘Competencies and conditions for co-production in research partnerships: A framework’ was published as a working document to be applied, reflected on and adapted as more learning was embedded.^32^

### Phase 4: working with community researchers to assess the accessibility and use of the framework

To further understand the potential utility of the framework for research teams, partners within each country worked with community researchers to gather their perspectives on the concept. Community researchers across countries identified that this competency framework would act as a:

- Reference point for the competencies and partnerships in the communities.
- Tool for planning and coordination among community researchers and between them, researchers and wider communities.
- Tool for monitoring and evaluation of their practices and outcomes in working both with communities and researchers.
- Tool to help assess and identify best practices by competency.
- Tool to assess change in competencies from activities that happen in the communities.

Some community researchers and implementing partners discussed how ongoing reflexive discussions could complement the use of the framework and support its evolution and utilisation within specific programmes/practices.

Some of the challenges and opportunities of using the competency framework with community researchers raised by researchers and implementing partners included the need for translation into the local language and/or talking through each competency in a reflexive session; validation workshops to adapt to context, providing practical and simplified examples to enhance accessibility; using the framework to track progress within the partnership as a mechanism to hold researchers accountable to CBPR principles, to ensure mutual capacity strengthening and power sharing; having simplified quality indicators aligned with each principle and clearly demarking competencies that apply to community researchers and academic researchers or implementing partners. These discussions resulted in streamlining the cross-cutting competencies (with embedded conditions) for ease of applicability.

### Phase 5: developing cross-cutting competencies and conditions

Following engagement with community researchers, consortium members read across all the findings and the core authorship group (BA, WA, RS, JAQ, NM, IC, KO, SR, LO and LD) synthesised the feedback to produce seven cross-cutting competencies and conditions which are presented in the results. To enhance understanding and applicability, a table of example activities and evidence sources from across the ARISE partnerships is displayed against each competency (see table 1).

### Patient and public involvement

The ARISE study was co-developed with community researchers who live and work in informal settlements in Bangladesh, India, Kenya and Sierra Leone. The ARISE hub has worked with community researchers as partners to set research questions, collect data, exchange knowledge and support people to exercise their right to health. Phase 4 above outlines how community researchers (the public) were involved in the development of the working framework and seven competencies presented in this manuscript.

### Findings

**Figure 2:**
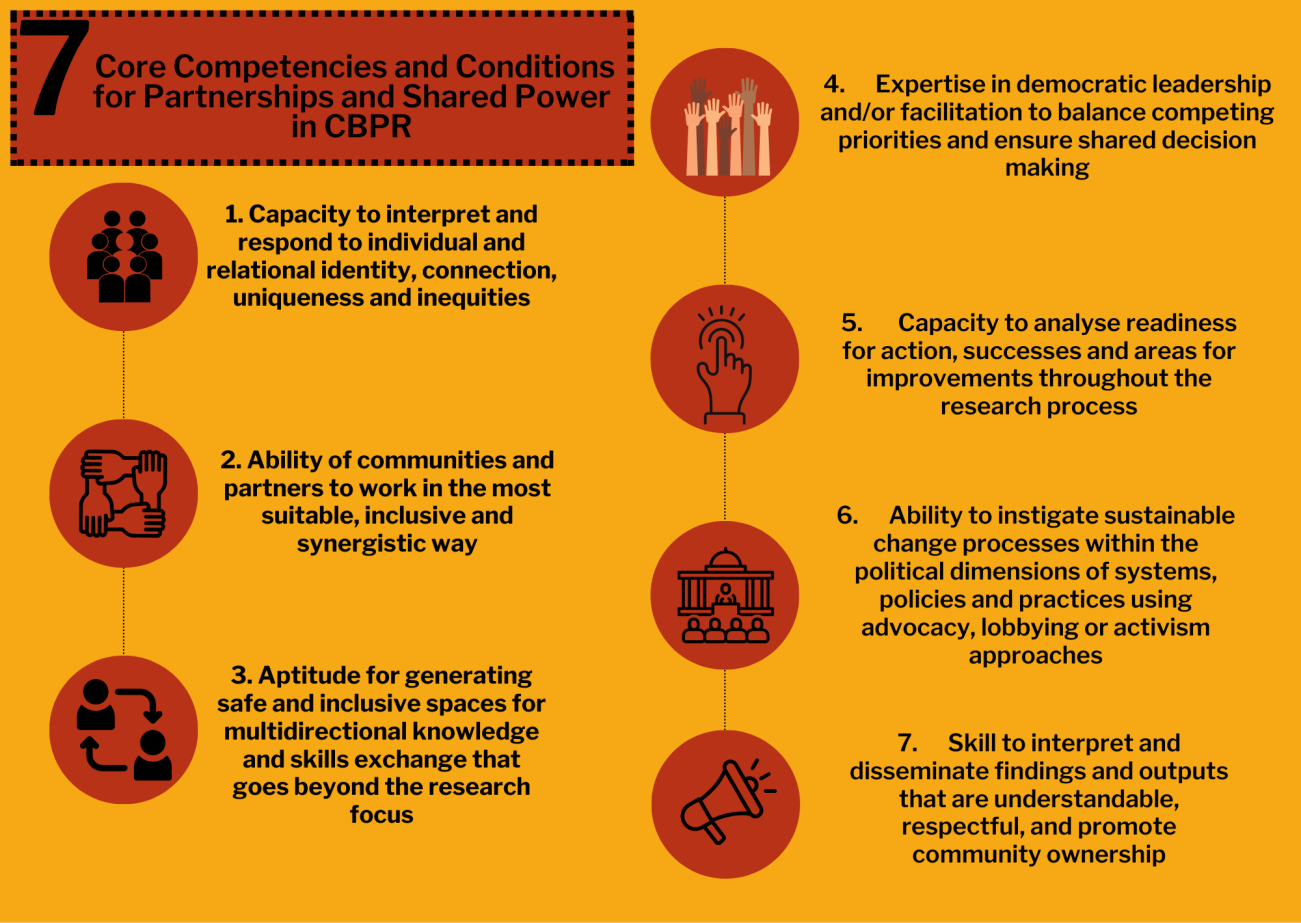
Seven core competencies to enhance the quality of community-based coproduction research partnerships. CBPR, community-based participatory research.

We present and discuss seven core cross-cutting competencies for coproduction in community-engaged research partnerships in figure 2 and below, drawing on the multistep methods described above. This section is structured as follows:

- An overarching description of the core competency and condition area.
- A list of individual and collective knowledge, skills or expertise associated with achieving the core competency or condition.
- A summary of likely outcomes for the project, community and partnership, should this competency be addressed.
- Examples of activities and sources of evidence presented in table 2 demonstrating application in a cross-country research project.

**Table 2:** Examples of activities undertaken to address the seven core competencies and conditions for CBPR and sources of evidence to demonstrate progress towards them from the ARISE consortium in Kenya, Bangladesh, India and Sierra Leone

Activities	Examples, evidence source or project achievements
**1. Capacity to interpret and respond to individual and relational identity, connection, uniqueness and inequities**	
Kenya: Marginalised populations such as people with disabilities, the elderly and child-headed households actively participated and collected their data by taking photos of their community. This helped build engagement in community activities, advocacy and connections with peers.	Blog published of reflections on the use of photovoice with disabled community members in urban informal settlements in Nairobi.^91^ Blog published by a community researcher with a disability on challenges faced in her community.^92^
Sierra Leone: Implementing partners brought together groups from three different informal settlements in Freetown to explore social inequalities, and their relationship to health and wellbeing within and across the settlements.	Reflexivity sessions were held with members of the three communities (community researchers and participants) which explored the partnership positionality throughout the research process at each stage.
**2. Ability to work closely with communities in the most suitable, inclusive and synergistic way**	
India: In Andhra Pradesh, waste workers used performative arts as a participatory tool for interaction between the community and governance actors. Performative artists worked with waste workers to cocreate several songs and skits, which were digitally recorded and performed in different settlements following training on street performance.	Process documentation of the workshop. Video recording of reflections by the performers and community members. Blog published on performative arts in India.^93^
Bangladesh: Research participants (vulnerable groups) and partners were identified based on pre-existing trusted relationships with community health volunteers who are trusted gatekeepers in the community and are well-informed about issues in the community.	Blog published on community researchers leading mapping of basic amenities in the community in Dholpur, Dhaka.^94^ Stories of change on working together with community researchers in Bangladesh.^95^ Reflexive meetings where community researchers were leading some capacity building training sessions.
**3. Aptitude for generating safe and inclusive spaces for multidirectional knowledge and skills exchange that goes beyond the research focus**	
Bangladesh: Discussion on health issues, service gaps with community members and governance actors (City Corporation CEO, Chief Health Officer, elected ward councillors) at Regional Learning Sharing Workshops helped build trust between marginalised communities and stakeholders and promote inclusive multidirectional knowledge exchange.	Blog published on regional learning-sharing workshops jointly organised with BRAC Urban Development Programme in three city corporations in Bangladesh.^96^
Sierra Leone and Kenya: Community researchers from Sierra Leone visited community researchers in Kenya to engage in cross-cutting learning and knowledge exchange to disseminate findings across contexts. Community researchers shared reflections and learnings on the different practices, and methods of transforming informal settlements while promoting health and well-being of residents.	Blog published on learning exchange visit between community researchers in Sierra Leone and Kenya.^97^
**4. Expertise in democratic leadership and/or facilitation to balance competing priorities and ensure shared decision-making**	
Bangladesh: Leadership training for Community Development Organisation members and active youth volunteers in urban informal settlements in Bangladesh helped improve leadership qualities so marginalised groups are better able to recognise their rights as citizens, analyse community needs, advocate for action and hold service providers and stakeholders accountable for their commitments.	Leadership training for community people in all five sites of ARISE Bangladesh from 11 December 2022 to 28 February 2023 (total participants: 159 (male: 22; female: 137).
Kenya: Community researchers were trained and supported to lead community prioritisation exercises. Capacity strengthening included how to guide a discussion and run such reflections, as well as how to present these priorities. Slum Dwellers International Federation members from the community who have skills in leadership mentored the community researchers on advocacy.	Youth leaders were incorporated within the national leadership group to do work around advocacy for human rights.
**5. Capacity to analyse readiness for action, successes and areas for improvements throughout the research process**	
India: In Mumbai and Ahmedabad, a health and well-being survey was undertaken to develop ‘discussion tools’ which were then used by Mahila Milan (a community women’s organisation) for thematic discussions with community members. These were used to plan action based on survey findings.	Pilot testing and reflection meetings. Translation of data into ‘discussion tools’ for easy readability and comprehension with tables, infographics and one-liners. Thematic focus group discussions. Letters to governance actors to demand action and accountability.
Sierra Leone: Following Geographic Information System (GIS) mapping, community researchers and researchers engaged in reflexive discussion sessions to discuss the political significance of boundaries, gaining insight into how it has shaped development and affects health and well-being in their communities.	Blog published on sharing experiences mapping urban marginalised spaces in Freetown, Sierra Leone.^64^
Bangladesh: An implementation partner assisted respective communities in developing community action plans (CAP) in Dhaka, Khulna and Satkhira, which helped communities to identify their priority health needs and developed action plans to address those. Based on the CAP findings, the implementation partner implemented community-led water, sanitation and hygiene (WASH) interventions. Another implementation partner facilitated community-led performance evaluations of two health centres through a community score card (CSC) tool in Rangpur city. One health centre management took immediate actions based on CSC findings for improving services.	CAP reports submitted to and endorsed by respective Ward Councillors. Respective community support groups used CAP report as a negotiation tool to demand services from other non-government organisations. Community representatives presented CSC findings to respective health centre authorities.
**6. Ability to instigate sustainable change processes within the political dimensions of systems, policies and practices using advocacy, lobbying or activism approaches**	
India: Marginalised residents from the communities demanded action on public health interventions in Mumbai through meetings conducted with governance actors and negotiated for actual on-ground changes in the primary healthcare facilities.	Quarterly review of changes in governance and accountability systems Minutes of meetings with governance actors
Siera Leone: Community members are represented on (District COVID-19 Response Committee), which allowed them to influence messaging to ensure it was appropriate for informal settlements and advocate for required resources such as handwashing stations.	Case study
**7. Skills to interpret and disseminate findings and outputs that are understandable, respectful and promote community ownership**	
India: Based on requests from waste worker communities in Delhi, Vijayawada and Guntur, a health and well-being survey was conceptualised and put into action. This created a community-owned repository of data on the health and well-being of waste workers to inform and enable demands for accountability based on these data.	An open access database of the waste workers on their health and well-being. Survey data—tools and approaches for analysis and action for different actors at different levels. Memorandum to governance actors by grassroot partners based on findings of the survey and identification of gaps.
Bangladesh: Community researchers actively contributed to competitions and conferences for sharing research findings. A community researcher from Bangladesh participated as a panellist in an organised panel session called ‘Accountability from below? Learnings from Participatory Research Processes on Water and Sanitation in Informal Urban Settlements’ at the seventh Global Symposium on Health Systems Research 2022, where she shared challenges related to WASH in the urban informal settlement where she is from and the local level accountability issues.	Video documentary presented in the seventh Global Symposium on Health Systems Research 2022 where a community researcher spoke about water and sanitation conditions and challenges faced by communities in Dholpur informal settlement, Dhaka.^98^
Sierra Leone: Researchers and community-based organisations collaborated to convene City Learning Platforms. These are events that bring together policy makers, communities, non-governmental organisations and researchers to discuss urban development topics informed by research. Community learning platforms are convened to feed information prioritised by community members into the City Learning Platforms.	Practitioner brief—Urban Health: From Local Community Action to a Healthy Freetown.^99^

ARISE, Accountability and Responsiveness in Informal Settlements for Equity; CBPR, community-based participatory research.

### Capacity to interpret and respond to individual and relational identity, connection, uniqueness and inequities

A participatory research partnership should be rooted in embracing shared identity, uniqueness and being open to learning and feedback from others. This includes an appreciation of how one’s own positionality shapes interactions with people, communities, and institutions and vice versa. In both cases, exploring identity takes time^8^ and requires competencies and conditions, including:

- A basic understanding of the concepts of intersectional inequities (social location based on social inequalities such as gender, age, race, education, dis/ability, religion, class, caste, language) including how they can manifest and change depending on context, time and space.^10 33^
- Being reflective on how communities or different partnership members may respond, view and receive researchers from outside their community, which is influenced by one’s social location.^34 35^
- Ability to recognise and respond to the ways in which power differentials and inequities shape participation and influence.^36^,^38^
- Ability to explore individual and shared identity using a variety of tools (culture, knowledge, strengths, talents, background).^34 39^
- Capacity to be reflexive, analysing potential strengths, bias, interpretations and limitations (individually and collectively).^3540^,^42^
- Openness to discuss issues of power that may have contributed to distrust between academically trained researchers and community partners.^43^,^45^
- Understanding of intragroup disparities and openness to making partners more culturally sensitive and open to alternative ways of thinking and ways of doing things.^39 43^
- Capacity to reﬂect personal, locational, institutional and structural power and to redress power imbalances to develop and maintain mutually respectful and dynamic partnerships with (as a researcher) and within communities (as a community researcher).^43 46^

‘Identity’ in this context extends beyond geography and depends heavily on community research partners’ perceptions of their shared experience, goals or emotional connectivity.^8^ People may belong to multiple communities (such as geographical, occupational or ideological) so working to identify the specific community of relevance for the work is important.^27 47^ This action requires capacities that promote the exploration of what identity means at different levels—both as an individual and relationally. Embracing individual diversity and recognising intersectional factors that impact on this shared identity is also important.

Being reflexive about identity, power, privilege, positionality and connectedness can often be uncomfortable, and, thus, avoided or deemed obsolete to the research aims.^3 48^ Evidence has shown that these ‘softer’ skills are mostly not addressed in participatory partnerships, which can lead to hidden or invisible inequities that can hinder trust.^36 49^ These skills have been found to lead to outcomes such as greater social cohesion or solidarity^50^; improved participation, communication, sensitivity and trust^29^; more opportunities and willingness for shared leadership and power; increased transparency of one’s positionality, strengths and challenges^48^; and increased empathy, compassion and humility.

Example activities that can help to develop these capacities include applying tools that explore power, identity and relations such as The Tree of Life,^51^ River of Life,^52^ using metaphors to explore identity in relation to others^53^ and applying Person-Centred Ways to Build Community.^54^ Table 2 provides examples of how reflexive data collection by community researchers across different informal settlements supported the exploration of power, stigma, language and partnership positionality in Sierra Leone and Kenya.

### Ability of communities and partners to work in the most suitable, inclusive and synergistic way

As communities are key to the research partnership, it is important to have the necessary skills to appreciate and work closely with(in) communities in the most suitable and inclusive way, including consideration of structures (physical spaces), institutions and people (political, social, historical, cultural, religious, others) and stakeholders (informal and formal actors that are influential or have power) that could contribute towards change.^8 55 56^

Competencies in this area are related to:

- Skills to examine and embrace how communities function.^14 57^
- Capacity to identify, understand and leverage community strengths and limitations.^8 56^
- Ability to identify and navigate social and structural assets/limits and governance structures that could support or hinder the research process or the inclusion of marginalised or vulnerable members.^58 59^ For example, being able to identify formal and informal governance structures in informal settlements that have an impact on health and well-being and working with the community to leverage these for improvements requires competencies in data collection, analysis and planning for change.
- Capability to assess motivations and involvement of gatekeepers who can at times silence marginalised voices or raise them.^36^
- Commitment to understanding the realities facing communities affected by the research area.^60^

Outcomes of developing and using these skills include more comprehensive and coordinated responses than any single stakeholder could achieve; generating networks and links across communities contributing to sustainability; increased resource base and optimisation; enhanced contextual readiness for research implementation^61 62^; improved impact on local policy and enriched understandings of the strengths, needs, priorities and health concerns of communities, organisations and health system; potentially leading to reﬁned and new research questions.^61^

Examples of activities that can support working closely with communities in the most suitable, inclusive and synergistic way include undertaking asset-based community development assessments^8^; applying governance assessments like ‘Governance diaries’ to identify informal and formal networks that could support action; having each research partnership member reflect on the strengths, resources and potential liabilities they and their organisation brings to the work^56^; asset and problem identification process such as Geographic  (GIS) mapping (remote/in person), walking and windshield tours,^63^ which involve walking or driving around the neighbourhood, documenting observations and impressions, or using a checklist to indicate assets or risks.^64^ Table 2 presents examples from ARISE countries of the mechanisms used to develop these competencies including using performing arts in India and working with trusted gatekeepers to identify marginalised people in Bangladesh.

### Aptitude for generating safe and inclusive spaces for multidirectional knowledge and skills exchange

This competency is about strengthening the capacity of the research partnership, both at the individual and collective level, for meaningful engagement in the research. Central to this is the valuable exchange of knowledge and skills within and across the research partnership and the identification of any knowledge gaps that could be filled by involving other partners, community members or investing in training. The competencies associated with this principle are focused on recognising different forms of knowledge and skills and embracing different learning styles and needs. Undertaking and monitoring capacity needs assessments as a partnership and developing individual and collective capacity strengthening plans will aid this process.^11 38^

When focusing on a specific research area, it is vital to recognise that research extends beyond the individual partnership members, to the immediate and larger contexts in which families live and work.^8 49^ Therefore, a key reflection of this competency is that not all capacity needs will have a direct link to the research or project but still have individual value which is important to recognise. For example, members of the research partnership may wish to develop skills such as sharing their views on what needs to be improved, applying for jobs or additional grants in areas not related to the current research area or undertaking further education. These should also be included and valued as a means of growing and maintaining the partnership and supporting individuals within it.

Competencies and conditions to consider include:

- Appreciation of the value of different forms of knowledge; experiential, contextual, technical, political, relational, historical and emotional.^65^
- Ability to identify one’s own capacities and skill gaps and those of peers.
- Openness to understand, listen to, and learn from people who might be different to yourself.
- Capacity to assess and communicate the immediate, potential, applied and realised value of social learning: ‘a process of social change in which people learn from each other in ways that can benefit wider social-ecological systems’ (Reed *et al*, p.3, Wenger *et al*).^66 67^
- Capacity to share knowledge in different ways that are accessible, relevant and tailored to the context.^68^
- Skills to assess and support all partners to engage in a process of investigating, sharing and reﬂecting on what does or does not work in their practice, as well as on how learning together contributes to making a diﬀerence.^18^

Outcomes from examining and addressing capacity needs include an enriched understanding, and mechanisms, to transparently discuss and address the strengths, needs, priorities, limitations and concerns of communities, organisations and systems.

Activities to meet this include developing a capacity strengthening strategy that identifies and evolves personal, organisational, physical, institutional and governance assets and strengths across research partners, monitored by documenting how different assets are shared and exchanged and measuring changes in capacity; and supporting community researchers to attend and share at conferences. Individual and collective capacity strengthening can be monitored using self-assessment, the ‘most significant change’ exercise or Ripple Effect Mapping.^69 70^ Table 2 demonstrates how Sierra Leone and Bangladesh teams facilitated spaces for multidirectional learning.

### Expertise in democratic leadership and/or facilitation to balance competing priorities and ensure shared decision-making

This competency relates to the continuous examination and adjustment of CBPR partnership governance and organisational structures that could support democratic practices for joint decision-making, leadership, information and power sharing throughout the research process. It also considers the democratic participation of communities, for example, through community advisory boards.^71^

Competencies and conditions to consider include:

- Ability to assess ‘goodness of ﬁt’ in terms of attitudes, beliefs and values for the compatibility and suitability of the partnership for the proposed CBPR project.^43^
- Expertise in balancing and discussing competing interests between community researchers, the broader diverse community, academic researchers and supporting organisations.
- Capacity to uphold common values, practices and behaviours that contribute positively to partnerships.^29^
- Being flexible, accommodating (open and respectful), compromising (to achieve consensus) and committed to moving the partnership and project forward.^59^
- Awareness of potential obstacles or enablers of strong partnerships, such as structures (ie, governing bodies, associations, policies), processes (ie, consenting or approval, procurement and allocation of resources), communication exchange, decision-making, leadership inﬂuences, pace and timelines.^14^
- Proficient leadership skills—developing the appropriate connections, motivating and inspiring others.^61^
- Democratic leadership and/or facilitation.^72^
- Understanding and ownership of varying roles within a group; catalyst, facilitator, colearner and/or consultant.^27^
- Focus on sharing information, decision-making power, resources and support.^71^

Key outcomes include establishing and implementing a shared vision of the research and its aims that is trusted and aspired to by all partners; increased and inclusive participation; strengthened relationships within the partnership and with communities that are likely to add sustainability to actions.

Activities include jointly producing a set of operating procedures such as ground rules and a memorandum of understanding; shared vision and associated goals; partnership communication mechanisms such as shared agendas and notes and a Code of Research Ethics and Safeguarding from the onset of the project.^4971 73^,^75^ Joint decision-making can be measured using qualitative and quantitative data to provide a comprehensive assessment and understanding of group development, function and impact which is evidenced by organisational structure subcommittees, rules, planning mechanisms, leadership stability and renewal policies.^71 76^ Table 2 presents ways that the partnerships in ARISE have strengthened leadership capacity and the ability to conduct and agree on community priorities in Bangladesh and Kenya.

### Capacity to analyse readiness for action, successes and areas for improvements throughout the research process

CBPR involves a cyclical, iterative process. At each of the research stages, the partnership should have the capacity to assess the community ecosystem (motivations and capacities of community stakeholders), sociocultural, economic and political conditions (national/regional/local elections, any risks to community participation), partner readiness and safety for taking action (assessing risks for individual involvement of partnership members is key to safeguarding) and have the capacity to closely monitor successes and areas for improvement so that micro changes are captured and learnt from.^43 77^ Capacities for this include:

- Capacity to assess ‘readiness’—the degree to which a community is prepared and safe to take action on an issue.^43 74^
- Understanding of diverse communities, the challenges they have experienced related to the health or social problem being addressed and the solutions they have tried.
- Listening, appreciating and acting on community thoughts, feelings, experiences and differences, from different perspectives to generate ideas for how to solve the problem.^78^
- Openness to design and test the different ideas within communities to solicit immediate feedback.^78^
- Ability to analyse successes and areas for improvements, to reflect on one’s experience and to assess the arguments and motivations of other stakeholders.^36 77^
- Examine ethical practice and safety during research process.^74 79 80^

The outcomes from strengthening these conditions and competencies include better understanding and use of research processes that lead to change; project evolution that is responsive to context, change and reflection; strengthened capacity to lead future research using a systematic and iterative approach; more accurate identification of community priorities and information; strengthened existing and new forms of community organisation to address priority issues and increased capacity of researchers to understand and respond to context and changing priorities of communities.

Activities and ways of measuring this core competency include:

- Engaging in joint problem-solving activities to prioritise and translate evidence into understandable formats that can be used to shape action, while considering safety and ethical practice.^80^
- Developing realistic action plans that identify gaps, causes of gaps, discrepancies and resolutions.^13^
- Modifying actions as necessary based on analysis of field notes and assessments following implementation at each stage of the cycle. This ensures that each subsequent action considers feedback from prior actions and that the result captures both successes and limitations.
- Engaging in learning cycles of action and reflection to inform revisions to the plans (actions and indicators).^36^
- Regularly revisiting monitoring indicators to ensure that they are updated to reflect the increasing complexity of the research as the programme matures.
- Facilitate coanalysis working sessions that support the research team to engage with research data to identify challenges that should be project priorities.
- Use planning models such as the PRECEDE-PROCEED model which provides a structure that supports the planning and implementation of health promotion or disease prevention programmes.^81^

Table 2 discusses ways that teams in India, Bangladesh and Sierra Leone generated actions that responded to the context.

### Ability to instigate sustainable change processes within the political dimensions of systems, policies and practices using advocacy, lobbying or activism approaches

CBPR emphasises integrating knowledge gained through the research partnership into sustainable interventions, practices and policies. To achieve this, partnerships may engage in activism and advocacy as well as research. Competencies and conditions for consideration are related to strengthening power, advocacy and influencing ability:

- Policy and advocacy skills within study sites, including extending community voices (or having community representation) in policy-making and influencing policies and practices aimed at improving health and well-being.
- Knowledge of how to frame issues, engage different audiences and promote belief that action can lead to change.^77^
- Ability to identify or develop group lobbying power to influence change in policy or policy processes.^58^
- Commitment to the integration of research results with community change efforts.
- Media or other forms of active engagement skills and relationships as appropriate to each specific context.
- Capacity to ensure regular and effective communication with practitioners and service delivery organisations to ensure that evidence influences the uptake and design of programmes.
- Understanding of the difference between programmes and policies, and the steps involved in developing a policy and/or advocacy campaign.^82^
- Knowledge of how a bill becomes law and who the major players are in decision-making.
- How to identify supporters and opponents.
- Creating a joint interpretive forum for sharing research knowledge.
- Understanding of the change process and awareness of the potential effects of politics on outcomes.
- Awareness of the effects of data and actions on the system in which research is involved.

Potential outcomes include generating public support for research; creating a critical mass for social change; minimising duplication of effort; people and institutions feeling empowered to take informed action and policy or practice change.

Ways to achieve the above competencies include mentoring community members to take a leadership role in the partnership and advocate for the health issues in the broader community^83^; providing training and technical assistance to enhance the capacity of community members to engage in the policy change process; mapping pre-existing power relationships—for example through power mapping, a tool to analyse power to shape a campaign strategy—to understand facilitators and hindrances to change; and active communication with policy-makers and strengthening alliances with other advocates. Table 2 outlines how communities in India and Sierra Leone implemented advocacy and lobbying skills to make changes in their communities.

### Skills to interpret and disseminate findings and outputs that are understandable, respectful and promote community ownership

Sharing findings with the broader community in an accessible and respectful way and providing opportunities for everyone to be involved in dissemination strategies such as publications and presentations at the local, state, district/county and national levels is important. Capacities and conditions to be considered include:

- How to identify audiences that need to receive the information and outputs.
- Interpretation of findings in ways that are understandable, respectful and where community ownership of knowledge is strongly recognised.^84^
- Capacity to purposefully develop and share new knowledge, products and resources that can inform policy and practice.
- Developing education materials outlining the key changes brought about through the CBPR process.
- Skills to engage in networking and the sharing of findings.
- Awareness of the need to adapt findings based on validation processes or suggestions from stakeholders where understandings may be misrepresented in initial research data.^85 86^

Outcomes related to the effective dissemination of new knowledge and actions include public interests are represented in the decision-making process; community residents perceive the value of engaging the CBPR process; changes from the CBPR process are embedded in networks and policy/practice and power is shared through joint development of publications and presentations which lead to new opportunities for the research partnership.

Activities to strengthen this core competency include developing a stakeholder communication mapping/matrix to inform the dissemination plan; using participatory methodologies to disseminate and validate research findings that draw on local community assets such as drama, art and storytelling and; developing and regularly reviewing a clear dissemination plan at project inception with inputs from all members of the research partnership. Table 2 provides some examples of how research findings were shared by community researchers in local, regional and global spaces of influence from three ARISE countries.

### Strengths and limitations

Limitations of our process include a restricted time frame for the scoping review and mixed levels of inputs from partners, including community researchers, which was determined by their availability, priorities and capacities at the time of our review. However, the value of the research was the ability to develop reflections and learnings over several years, including examples from the field and from multiple positionalities and experiences.

We understand that the seven competencies are complex and numerous, which may make it challenging to apply in projects with time or resource constraints. In addition, while we developed the competencies throughout the ARISE programme, we were unable to apply them from the beginning of the project, which is something we would recommend doing. We suggest getting to know the competencies and organising a workshop with partners, either collectively or separately with shared feedback (to ensure fairness), to determine which competency areas are the most important and how they can be addressed by leveraging the strengths of all partners to ensure fair participation.

In addition, when considering the competencies and conditions suggested here, it must be recognised that contexts change rapidly due to crises, emergencies and political situations such as the COVID-19 pandemic in 2020, or the forcible eviction of informal settlement residents in Dhaka city where ARISE was working. Therefore, priorities may shift and adapting quickly to the needs of the partnership and associated stakeholders will be important. This is why we recommend a continual and embedded approach to assessing the quality of CBPR partnerships with committed opportunities for reflexivity that respond to the seven competency and condition areas highlighted above.

Reflexivity refers to the continuous process of self-reflection that researchers engage in to generate awareness about their actions, feelings and perceptions.^35^ Inherent to this process is examining power, power-sharing principles and shared decision-making. Creating individual and collective spaces to reflect on the seven competencies will support identifying competency and condition challenges and promote discussions on how best to address these. For example, within the ARISE project, partners across the countries embedded reflexivity sessions, as shared in table 2.

### Future research

The seven competencies have the potential to be applied by a variety of research approaches that centre coproduction and equitable research partnerships, including but not limited to; learning health systems, participatory action research, quality improvement cycles, human-centred design, people-centred health systems and CBPR. The seven competencies were produced to advance the science of CBPR and coproduction research by first cocreating a baseline of competency and condition areas that are likely to need attention within CBPR partnerships. The next step will be to test their utility in terms of improving the quality, transparency and rigour of coproduction research.

It is anticipated that these competencies will evolve through application by different users such as community and academic researchers, implementing organisations, supporting institutions and health systems actors. Based on these experiences, tools and associated guidelines will require development to ensure the language and areas presented are accessible and have meaning to all research partners including our community partners.

We encourage others to test these competencies in their research contexts and share their learnings to further refine and improve the framework in the future.

## Conclusion

As the global health research community strives for more equitable research partnerships that prioritise local knowledge generation and leadership, it is important to examine and be transparent about competency gaps and limiting conditions that hinder meaningful participation or enable dominance by some partners. By acknowledging and addressing these gaps, we can contribute to decolonising research at the project level and begin to shift inequitable cultural and power dynamics within research practice. The seven competency areas outlined here can serve as a guide to exploring ways to strengthen the competencies and conditions of all partners, establish quality benchmarks, and gather evidence to improve the quality of CBPR projects.

We call for more focus on examining competency needs and sociopolitical conditions within coproduced research that engages the public. Now, more than ever, it is critical, as we see global shifts in technology, urbanisation, globalisation, populism, artificial intelligence, immersive virtual experiences and social structures. These rapidly changing contexts and connection mechanisms call for new approaches to community-engaged coproduction research and learning of novel ‘hard’ and ‘soft’ skills. Researchers engaging with the public seek to connect with people and society to discover new knowledge, inspire new ideas and create solutions. To do this well, we must acknowledge and examine what skills, expertise and conditions will support optimal and equitable coproduction research practice and outcomes. The seven competencies offered here can support partnerships to begin and expand this practice.

## Supplementary material

10.1136/bmjgh-2024-015497 Uncited online supplemental file 1

## Data Availability

Data sharing is not applicable as no datasets were generated and/or analysed for this study.
