# Supplementary material for: Seven core competencies and conditions for equitable partnerships and power sharing in community-based participatory research
Source: BMJ Glob Health. 2024 Nov 17;9(11):e015497. doi: 10.1136/bmjgh-2024-015497 (PMC11574420; doi:10.1136/bmjgh-2024-015497)
Supplement: Uncited online supplemental file 1 [file bmjgh-9-11-s001.docx]

# Reflexivity Statement

This study was co-developed by researchers, implementers (e.g. health workers, non-government development partners), and community researchers working in ‘The Accountability and Responsiveness in Informal Settlements for Equity’ hub (ARISE). ARISE applied community-based participatory research (CBPR) in Bangladesh, India, Kenya and Sierra Leone to support people living and/or working in urban informal settlements to claim their rights to health. The authorship list includes input from researchers, at different stages of their careers, from each country within the ARISE consortium. All authors were involved in progressing the study findings at different stages (conceptualisation, literature review, review of synthesised findings, adding experiential evidence and recommendations and writing this manuscript) over a three-year process. In addition, we sought input from community researchers which is discussed in step four of the methods. The findings from this research have been applied locally by partners in each country setting (see Table 1) and promoted through a CBPR-focused subgroup within the ARISE consortium that consisted of partners from all countries. With a commitment to gender diversity and inclusion, the ARISE Consortium ensured a comprehensive representation of perspectives throughout the research process and partnership. Community researchers were carefully selected from diverse backgrounds, including individuals of different genders, ages, races, religions, ethnicities, and disability statuses in each partner country. The group of authors also reflects a range of backgrounds, with all lead authors being women. Knowledge exchange and discussion that aimed to support capacity strengthening and exchange for CBPR practice of all partners was embedded in the form of discussion workshops, presentations, shared resources and written comments. We have collectively agreed joint first and last authorship to reflect input and to align with authorship guidelines.
